# Living in mixed species groups promotes predator learning in degraded habitats

**DOI:** 10.1038/s41598-021-98224-0

**Published:** 2021-09-29

**Authors:** Douglas P. Chivers, Mark I. McCormick, Eric P. Fakan, Randall P. Barry, Maud C. O. Ferrari

**Affiliations:** 1 Department of Biology, University of Saskatchewan, Saskatoon, SK S7N 5E2 Canada; 2 ARC Centre of Excellence for Coral Reef Studies, James Cook University, Townsville, QLD 4811 Australia; 3 Department of Biomedical Sciences, WCVM, University of Saskatchewan, Saskatoon, SK S7W 5B4 Canada

**Keywords:** Ecology, Zoology

## Abstract

Living in mix-species aggregations provides animals with substantive anti-predator, foraging and locomotory advantages while simultaneously exposing them to costs, including increased competition and pathogen exposure. Given each species possess unique morphology, competitive ability, parasite vulnerability and predator defences, we can surmise that each species in mixed groups will experience a unique set of trade-offs. In addition to this unique balance, each species must also contend with anthropogenic changes, a relatively new, and rapidly increasing phenomenon, that adds further complexity to any system. This complex balance of biotic and abiotic factors is on full display in the exceptionally diverse, yet anthropogenically degraded, Great Barrier Reef of Australia. One such example within this intricate ecosystem is the inability of some damselfish to utilize their own chemical alarm cues within degraded habitats, leaving them exposed to increased predation risk. These cues, which are released when the skin is damaged, warn nearby individuals of increased predation risk and act as a crucial associative learning tool. Normally, a single exposure of alarm cues paired with an unknown predator odour facilitates learning of that new odour as dangerous. Here, we show that Ambon damselfish, *Pomacentrus amboinensis*, a species with impaired alarm responses in degraded habitats, failed to learn a novel predator odour as risky when associated with chemical alarm cues. However, in the same degraded habitats, the same species learned to recognize a novel predator as risky when the predator odour was paired with alarm cues of the closely related, and co-occurring, whitetail damselfish, *Pomacentrus chrysurus.* The importance of this learning opportunity was underscored in a survival experiment which demonstrated that fish in degraded habitats trained with heterospecific alarm cues, had higher survival than those we tried to train with conspecific alarm cues. From these data, we conclude that redundancy in learning mechanisms among prey guild members may lead to increased stability in rapidly changing environments.

## Introduction

The world is experiencing an unprecedented decline in biodiversity^1–3^. This phenomenon is particularly evident in highly diverse habitats such as coral reefs, which are now among the most threatened ecosystems on the planet^4,5^. Recent increases in global temperature have resulted in hundreds of square kilometers of dead and dying corals^6^. This largescale death and decline in diversity, is concurrent with a change in community composition and ecological functioning^7,8^. For example, the decline in fish populations has been linked to alterations in predator–prey dynamics^9^. Specifically, it appears that risk perception and swim physiology for some species is fundamentally changed in dead and degrading coral environments^10,11^.

In the most comprehensive study to date, Ferrari et al.^12^ documented that several species of damselfish (Pomacentridae) lose their ability to respond to olfactory cues that normally indicate risk. When corals die, their skeleton is taken over by algae and cyanobacteria, which subsequently modifies the ‘olfactory landscape’ of surrounding habitats, incidentally rendering alarm cues ineffective in some species^13^. Interestingly, not all species of damselfish are affected^12^. However, for those that are affected, the implications of the loss of alarm cues are considerable, given these chemicals released from the skin of damaged fish, mediate an array of behavioural and morphological defences, including the learning and recognition of novel predators^14,15^.

The failure to learn to recognize novel predators has tremendous survival costs for newly recruited predator naïve fish arriving at the reef. For example, Juvenile Ward’s damsels (*Pomacentrus wardi*) taught to recognize the sight or odour of common reef predators have up to eight times higher survival over the first few days on the reef compared to those that were not trained^16^. Likewise, Ambon damselfish (*P. amboinensis*) that underwent predator training had a 3.5 times greater survival upon recruitment to the reef^17^.

Coral reefs are highly diverse ecosystems, granting them considerable redundancy and impact reduction when facing change. For example, normally, juvenile damselfishes learn to identity novel predators when they detect conspecific alarm cues paired with the sight or smell of a novel predator^18^. This is a powerful learning mechanism, with a single conditioning trial facilitating predator learning. However, in a recent study, Chivers et al.^10^ showed that Ambon damselfish, held in water that contained dead coral, lost their ability to learn predators via association with conspecific alarm cues. However, these fish could learn socially to recognize predators from Nagasaki damselfish (*P. nagasakiensis*), a species that appears to be unaffected by coral degradation. Clearly, fishes in high diversity habitats can gain tremendous advantages by accessing information held by prey guild members^19^. It is precisely this type of predator learning redundancy that creates resilience to habitat degradation.

In this study, we address a broader question about the potential of learning redundancy in coral reef systems. We ask whether a species that is affected by coral degradation, in this case the Ambon damselfish, *Pomacentrus amboinensis*, can learn the identity of unknown predators in degraded coral from direct conditioning with alarm cues from a closely related species, the whitetail damselfish (*P. chrysurus*), whose alarm cues are resistant to the effects of coral degradation^12^.

Cross-species responses to alarm cues in mixed species assemblages are common^20^ and are often based on innate recognition of chemically similar alarm cues among closely related species^21,22^. Cross-species responses to alarm cues can also occur between distantly related prey species that share common habitats and predators^23^. In one of the pioneering studies in this field, Chivers et al.^24^ showed that brook stickleback (*Culaea inconstans*) respond to minnow (*Pimephales promelas*) alarm cues and can use minnow alarm cues to learn the identity of pike (*Esox lucius*) as a predator. Similarly, in the current experiment, we conditioned predator-naïve Ambon damselfish to recognize the odour of predatory dottyback (*Pseudochromis fuscus*) as risky by exposing them to conspecific alarm cues (positive control), heterospecific alarm cues or seawater (negative control) in tanks containing water from either healthy or degraded corals. We followed this laboratory investigation by directly testing whether fish that have undergone survival training with conspecific and heterospecific alarm cues in healthy versus dead coral environments have higher survival when stocked onto the reef. Assessing survival in the wild is the best way to test the importance of learning redundancy.

## Methods

All research herein was conducted in accordance with the James Cook University Animal Ethics guidelines with approval from the JCU Animal Ethics Committee (approval A2408). The study was carried out in compliance with the ARRIVE guidelines**.**

### Test species and experimental cues

Laboratory and field studies were conducted in November 2017 at Lizard Island (14° 40′ S, 145° 28′ E), on the northern Great Barrier Reef, Australia. The Ambon damselfish and Whitetail damselfish are common within coral reef fish communities of the Indo-Pacific. For the current study we collected fish at the end of their larval phase using light traps. Light traps, deployed overnight, were moored at least 30 m from the nearest reef edge. Captured fish were brought to the laboratory in 60-l tanks at dawn, sorted by species and transferred to 35-l tanks of aerated flow-through seawater. Water was pumped directly into the research station distribution system from an inlet pipe approximately 100 m from shore on a sand flat. The closest reef was approximately 20 m from the inlet pipe. Fish were fed three-times a day with newly hatched brine shrimp (*Artemia* *nauplii*) for several days prior to beginning the experiments. The collection location of our traps away from the reef meant that our test fish have no experience with specific predators that awaited them upon settlement. Previous research on juvenile damselfish has found marked antipredator response to alarm cues prepared from conspecifics and some heterospecific damselfish^25^. This antipredator response is characterized by reduced foraging and activity, and increased shelter use^26^.

Nineteen dottybacks, *Pseudochromis fuscus*, three moonwrasse, *Thalassoma lunare* and five lizardfish, *Synodus dermatognys* were caught by SCUBA using hand nets and clove oil. Sixteen dottybacks were used in experiment one. These predators were held in pairs in 15-l flow-through tanks but housed individually in mesh containers to reduce aggression. All predators were fed thawed squid once per day. Water flowing through these tanks came from one of two header 15-l tanks containing either a piece (~ 60 cm in circumference) of healthy, live *Pocillopora damicornis*, a hard, bushy coral commonly found at our field site, or an equal sized piece of dead-degraded coral that was encrusted with algae. The corals were collected from several sites around the Research Station. We did not quantify the amount of encrusted algae on the dead corals, however both coral types were changed several times over the course of the experiment. The header tanks were equipped with an airstone, and had constantly flowing fresh seawater at a rate of 1 l/min. Each header tank was plumbed in a way that allowed the overflow to enter four predator tanks. To prepare predator odours, we diverted the water flow from the header tank 2 h prior to collection to allow the accumulation of the predator odour in the tank. This water was used as our healthy or degraded coral predator odour.

The remaining three dottybacks, as well as the moonwrasse and lizardfish were used in experiment two. They were held in 35-l flow-through tanks, separated by species, and were used to generate predator odours for experiment two. As in experiment one, the water flow from the header tank was stopped for two hours to allow odour to accumulate in the predator tank.

Alarm cues (AC) used in experiments one and two were prepared fresh, minutes prior to use. Donor fish were euthanized via cold shock followed by cervical dislocation. For experiment one, we used a scalpel to make four cuts on both sides of three conspecific (*P. amboinensis*) or heterospecific (*P. chrysurus*) damselfish and then rinsed them in 20 ml of seawater. This AC solution was then split in half and 20 ml of water from either the healthy coral tank or 20 ml of water from the degraded coral tank was added to each solution, resulting in 30 ml of AC solution from healthy coral and 30 ml of AC solution from degraded coral. When we introduced 10 ml of the solution into our 2-l conditioning tanks (see below), we had a final concentration of 2 cuts/l of water, which is a standard concentration known to elicit overt antipredator responses^10^. For experiment two, we followed the same procedure, except we increased the number of fish donors and cuts, to account for having 15-l conditioning tanks, and maintaining a concentration of two cuts/l in the tank.

## Experiment 1: Learned predator recognition test in the laboratory

### Overview

We used an established protocol to test for learned recognition of predators^18,27^. Predator-naïve *P. amboinensis* juveniles were conditioned to recognize a predatory dottyback by exposing them to conspecific alarm cues (positive control), heterospecific alarm cues or seawater (negative control) paired with dottyback odour in tanks containing water from either healthy or degraded corals. This resulted in a fully crossed 3 × 2 design (see Table 1). In all cases, the coral water (live or degraded, see below for details) used for the predator odour and alarm cues preparation matched the coral water treatment at conditioning. The day after conditioning, we tested the fish for their response to predator odours alone, making sure that fish trained in degraded coral were tested in degraded coral and vice versa.

**Table 1:**
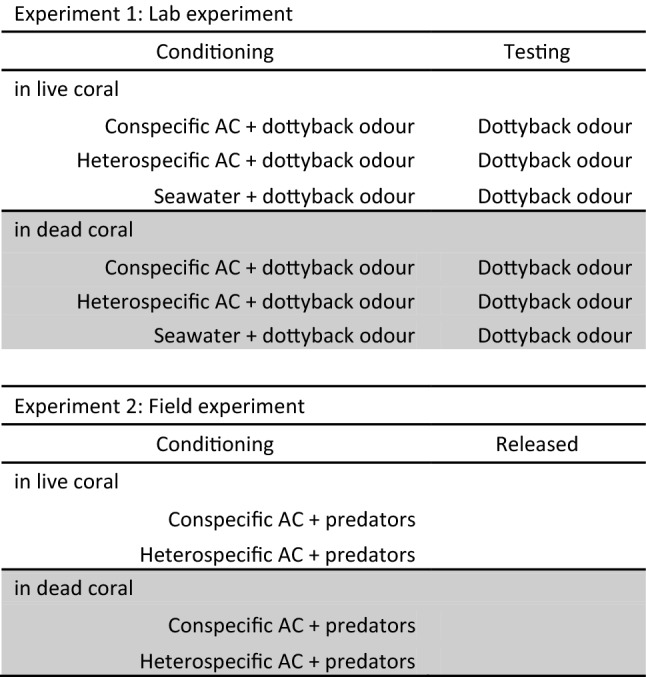
Summary of treatment groups in Experiments 1 and 2.

Conspecific AC: Alarm cues from *P. amboinensis*; Heterospecific AC: Alarm cues from *P. chrysurus*. Alarm cues and predator odours were prepared with water that matched their respective treatment (see text for details). For experiment 1, we conditioned prey and the following day quantified behaviour (changes in feeding and activity) following exposure to Dottyback odour in the laboratory. For experiment 2, we conditioned prey in live or dead coral to recognize the sight and smell of three common reef mesopredators (Dottyback, Moonwrasse, Lizardfish) in the laboratory and transferred them to patch reefs that were situated 4 m from the continuous reef edge. We quantified behaviour (bite rate, total distance moved, maximum distance ventured from the habitat patch and boldness) and survival.

### Conditioning phase

We placed groups of three juvenile fish into 2-l plastic conditioning tanks containing a sandy substrate and a piece of clean, bleached coral. The tanks had flowing seawater from four header tanks containing either live or degraded coral, in a setup identical to the one described for the predator odour. The coral header tanks had constantly flowing fresh seawater at a rate of 1 l/min, flowing into the conditioning tanks. Both coral types were changed every few days. The fish were placed in the conditioning tank 22 h before the start of conditioning. True conditioning procedure consisted of injecting 10 ml of conspecific AC, heterospecific AC or water, simultaneously with 20 ml of predator odour into each tank. The source of the predator odours were chosen randomly from the four healthy and four degraded predator odour sources available each day.

### Testing phase

One hour following the end of the conditioning phase, fish were moved individually into 2-l plastic tanks, equipped with a sand substrate, a piece of clean bleached coral (10 cm high) serving as a shelter, and an air stone, to which was attached a 1.5 m long injection hose. A 4 × 4 cm grid was drawn on the tank to facilitate data collection. Once again, each testing tank received flow-through water from a header tank containing live or dead coral, as described above. Each test tank received water at a rate of ~ 1 l/5 min. The fish were left to acclimate overnight and were tested the following day.

The bioassay followed established protocols^12,18^. In summary, the behaviours of each fish (number of feeding strikes and lines crossed, as measures of feeding and active swimming) were observed for 3 min before and after the introduction of a stimulus (20 ml of predator odour, of matching coral environment). Reductions in feeding and activity are both well-established antipredator responses^18,28^. All fish were tested for their responses to predator odours that were generated by different individuals from the ones used during the conditioning trials. Trials were randomized and performed blind.

## Experiment 2: In situ consequences of learned predator recognition

### Overview

Predator-naïve juvenile *P. amboinensis* were conditioned to recognize the smell and the sight of three common reef predators (lizardfish, moon wrasse and dottybacks) via exposure to either conspecific alarm cue, or heterospecific alarm cue in tanks containing water from either healthy or degraded corals, in a fully randomized 2 × 2 design (see Table 1). These species are common mesopredators of juvenile damselfishes on coral reefs^29^, particularly near the reefs used for our field experiments. Fish were then transferred individually to patch reefs in the wild, and their survival assessed over a period of 72 h.

### Predator conditioning

Two days prior to the start of the experiment, juveniles were batch tagged with a fluorescent elastomer^30^. This allowed us to distinguish them from new juveniles naturally recruiting onto our patch reefs. Then, groups of four fish were randomly allocated to one of twelve 15-l conditioning tanks, three tanks in each of our four treatment combinations. After an overnight acclimation, the fish were conditioned following established protocols^31^. Fish were exposed to the sight and smell of each of our three predators using alarm cues from conspecifics or heterospecifics (*P. chrysurus*), in water that had passed through a header tank containing either healthy live or dead-degraded coral. The conditioning protocol consisted of introducing 20 ml of alarm cue solution and 60 ml of each predator odour into the tank at the same time as a clear plastic bag containing the corresponding predator was placed into the tank with the prey. The predator remained in the tank for 1 min. After one min, the procedure was repeated using a different predator, until each prey was exposed to all three predators. This was repeated over five batches of 11–32 fish over a 5-day period, with every treatment equally represented across batches.

### Behaviour in the field

One hour following conditioning, fish were placed into individually numbered 1-l plastic clip-seal bags containing aerated water and photographed against a 1-cm grid to obtain a size estimate. Fish in bags were then placed in a 60-l container of seawater, covered in shade cloth and taken by boat to the edge of a shallow fringing reef. Fish were placed by divers (MIM, EF) on small numbered patch reefs (25 × 15 × 20 cm) made of clean bleached *Pocillopora damicornis*, a bushy hard coral and part of their naturally used settlement habitat^32^. A small cage (11-mm mesh size; 30 × 30 × 30 cm) was placed over the patches to allow acclimation to the reef without the threat of predation. Treatments were systematically placed down the reef to avoid any possible confounding with reef position. Patch reefs were 4 m apart and 4 m from the continuous reef edge.

The cages on the patch reefs were removed 40–60 min after fish were release on the patch after which the activity and space use of the fish was assessed using an established protocol^33^. All observations were conducted by a single observer (MIM) who was blind to the conditioning treatments. Briefly, fish behavior was assessed in situ over a 3-min period by an observer on SCUBA that was ~ 1.5 m away from the patch reef with the aid of a 4× magnifying glass. Four aspects of activity and space use were assessed: (i) bite rate; (ii) total distance moved (estimated from knowing the length of each reef); (iii) maximum distance ventured (Max DV) from the habitat patch; (iv) boldness. Boldness was assessed using a continuous scale between 0 and 3 where: 0 is hiding in hole and seldom emerging; 1 is retreating to hole when scared and taking more than 5 s to re-emerge, weakly or tentatively striking at food; 2 is shying to shelter of patch when scared but quickly emerging, purposeful strikes at food; and 3 is not hiding when scared, exploring around the coral patch, and striking aggressively at food^33^. This boldness measure has been shown to be repeatable (e.g. repeatability values of ~ 0.5 over a 2 h period;^34^. Three-min behavioural assessments have previously been found to be sufficiently long to obtain a representative estimate of an individual’s behaviour^34,35^.

### Survival monitoring

Fish on patch reefs were monitored three times per day for 72 h (7:00, 12:00, 17:00). On the rare occasion that other fishes settled to the occupied reefs, these were removed with a dip net at the time of census.

### Statistical analysis

#### Experiment 1

Because feeding and activity are related, we ran a correlation-based PCA to obtain a single synthetic score from the two behaviours. We performed this step to obtain a synthetic variable for the pre-stimulus data (to test for bias in baseline behaviour among the treatment groups) and for the proportion change in behaviour from the prestimulus baseline data. This proportion change was obtained as followed: ([post–pre]/pre). These scores were used as the response variable in subsequent univariate analysis.

We performed linear mixed model ANOVAs, testing the effect of coral environment (healthy vs. degraded) and conditioning treatments (water conditioning, conspecific alarm cue conditioning or heterospecific alarm cue conditioning) on the response variables. We introduced ‘conditioning tank’ a nested, random factor and we used type I SS appropriate for hierarchical models. This nested approach accounted for the dependence of all the fish conditioned in the same conditioning tanks, hence using ‘conditioning tank’, rather than ‘fish’ as the level of replication in the analysis. Data met parametric assumptions.

#### Experiment 2

The four behaviours (bite rate, total distance moved, maximum distance ventured and boldness) were combined into a single behavioural score using a correlation-based PCA. We then performed a 3-way nested ANOVA, testing the effect of coral environment (healthy vs. degraded) and conditioning cue (conspecific vs. heterospecific alarm cue) on the behavioural score of the fish. We introduced ‘conditioning tank’ as a nested factor (Type I SS), to account for the interdependence of the fish conditioned in the same tanks, making ‘tank’, rather than ‘fish’ the level of replication for conditioning. Survival was analysed using a Kaplan–Meier survival analysis.

## Results

### Experiment 1

We failed to find any bias among treatment groups in the baseline behaviour of the fish (3-way nested ANOVA, all *p* > 0.5, see Supplementary Table 1).

**Figure 1:**
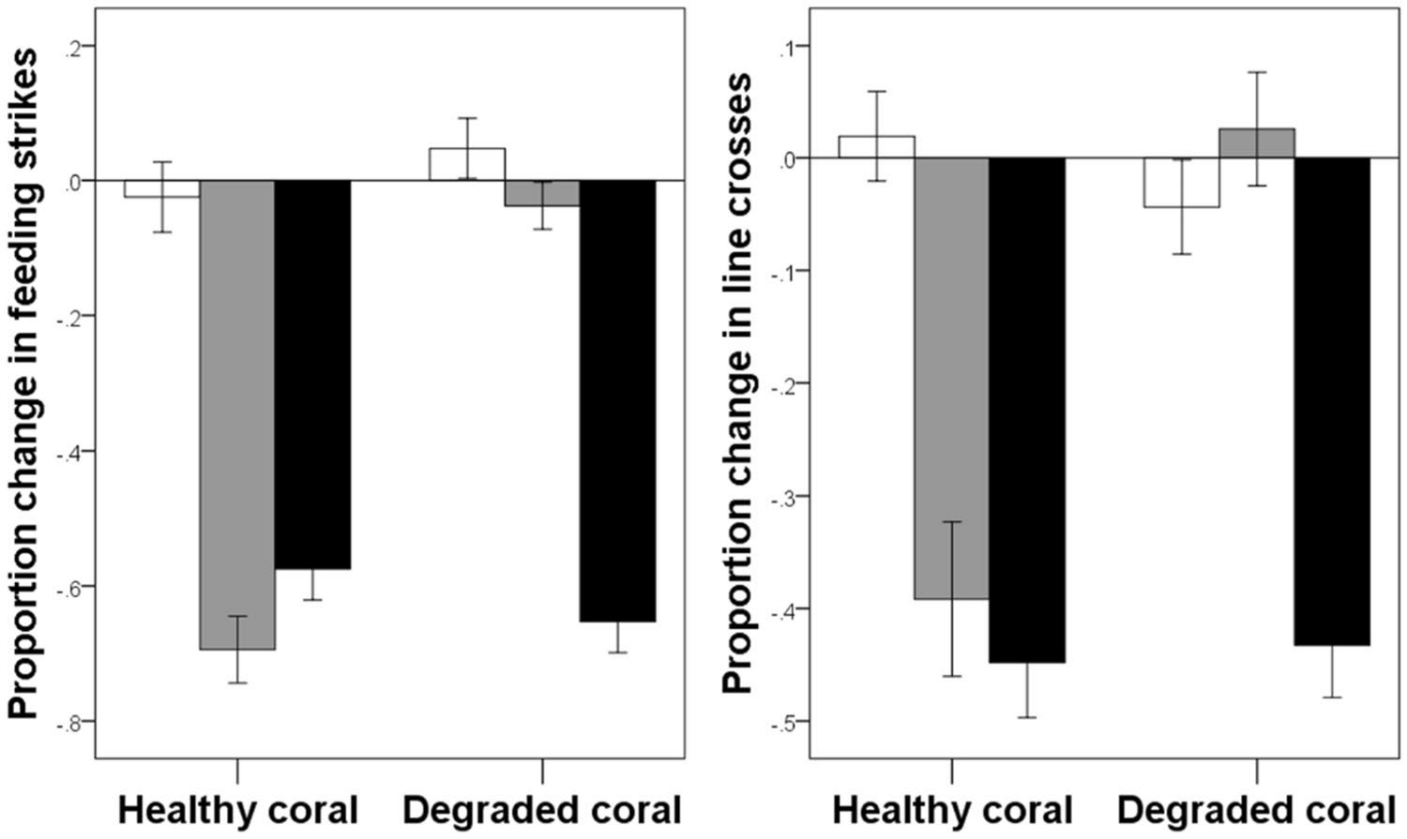
Mean (±SE) proportion change in feeding strikes (left) and line crosses (right) for juvenile *P. amboinensis* exposed to the odour of a dottyback. Juveniles were conditioned to recognize the dottyback odour by a pairing with water (white bars), alarm cues from conspecific *P. amboinensis* (grey bars) or alarm cues from closely-related heterospecific *P.* chrysurus (black bars).

The response of the fish to the predator was affected by an interaction between coral environment and conditioning treatment (F_2,76_ = 22.8, *p* < 0.001, Fig. 1, see Supplementary Table 2), meaning that the effect of conditioning was contingent upon the coral environment in which the fish were maintained. Fish maintained in water from healthy coral showed no response to the predator when pseudo-conditioned with water, but showed strong antipredator responses when the conditioning was performed with alarm cues from either conspecific or heterospecific donors (Tukey post-hoc comparisons: water vs. conspecific: *p* < 0.001, water vs. heterospecific: *p* < 0.001, conspecific vs. heterospecific: *p* = 0.96). Fish exposed to degraded coral water, on the other hand, differed in their responses to predator odour when conditioned with conspecific cues. Indeed, they showed no difference in response to the predator when conditioned with water or conspecific alarm cues (Tukey post-hoc comparisons: *p* = 0.99), while they only showed a strong antipredator response when they were conditioned with heterospecific alarm cues (water vs. heterospecific: *p* < 0.001, conspecific vs. heterospecific: *p* < 0.001).

### Experiment 2

**Figure 2:**
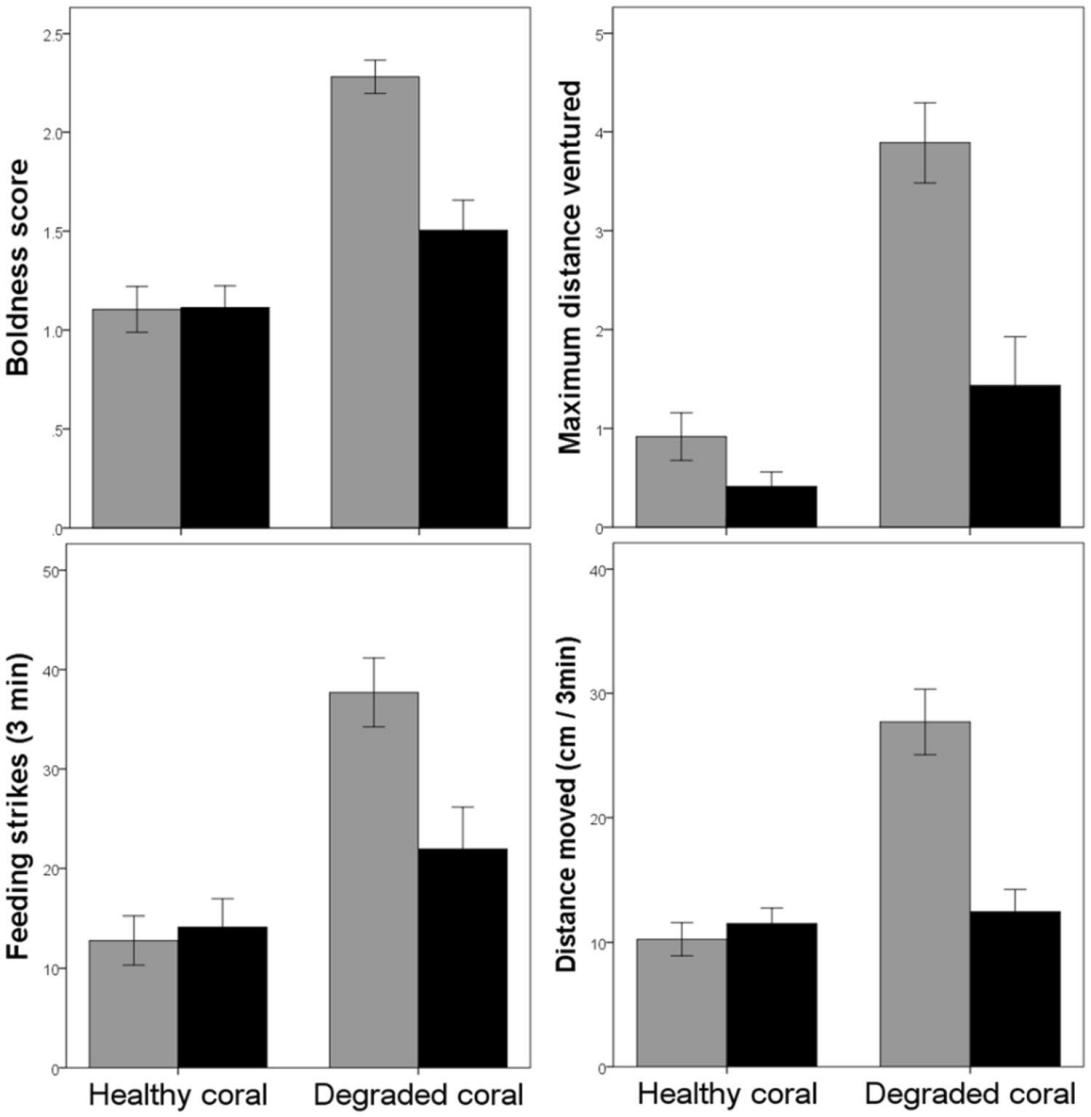
Mean (± SE) boldness score (top, left), maximum distance ventured (top, right), number of feeding strikes (bottom, left) and distance moved (bottom, right) for *P. amboinensis* in situ. Prior to deployment in the field, *P. amboinensis* were taught to recognize three common predators (lizardfish, wrasse and dottyback) using alarm cues from conspecifics (*P. amboinensis*, grey bars) or alarm cues from a closely-related heterospecific (*P. chrysurus*, black bars). The training took place in water containing cues from either healthy, live or dead, degraded *Pocillopora damicornis* coral.

PC1 scores captured 74% of the variance. The 3-way nested ANOVA revealed that the behaviour of the fish in the wild were contingent on the coral environment and the alarm cues with which they were trained (coral × cue: F_1,6.1_ = 11.5, *p* = 0.014, Fig. 2, see Supplementary Table 3). When *P. amboinensis* juveniles were trained to recognize the predators with alarm cues from *P. chrysurus*, their behaviour in the field was not affected by the coral training environment (*p* = 0.25). However, when they were trained with conspecific alarm cues, their behaviour was less conservative if they had been conditioned in degraded coral rather than in healthy coral (*p* = 0.011). In addition, in live coral, the behaviour of the fish did not differ among the two training cues (*p* = 0.95).

**Figure 3:**
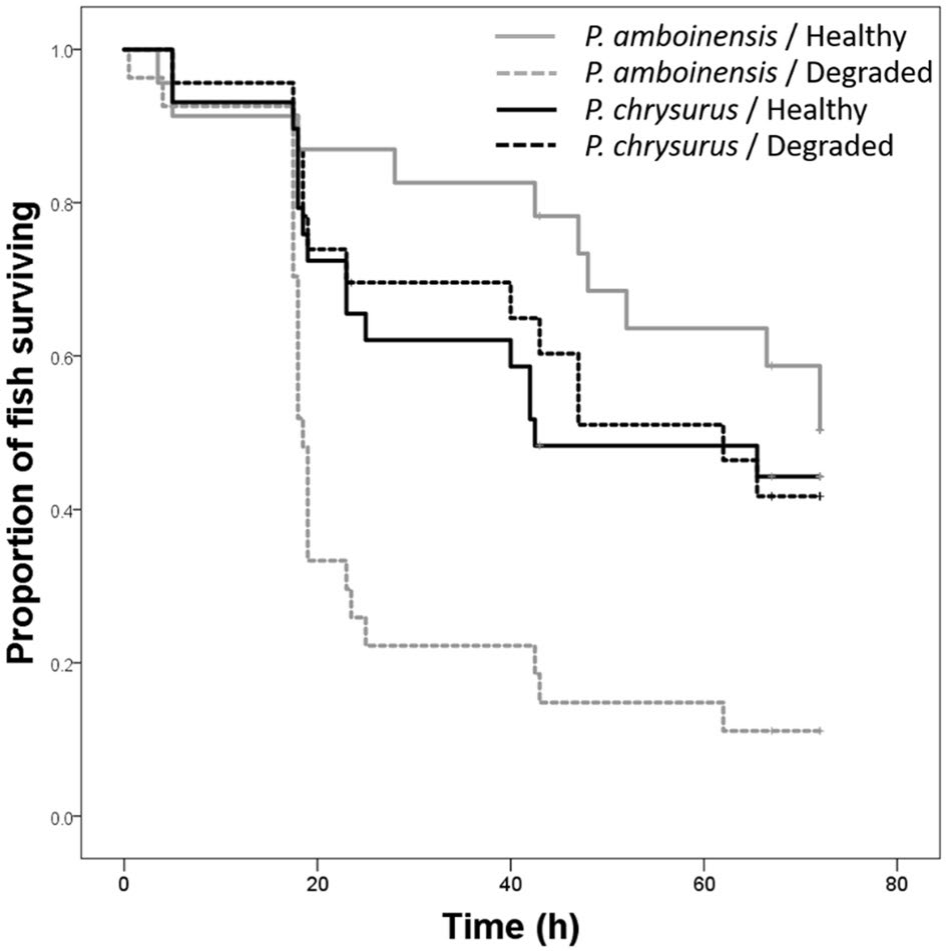
Cumulative proportion of *P. amboinensis* surviving over time in situ. Prior to deployment in the field, *P. amboinensis* were taught to recognize three common predators (lizardfish, wrasse and dottyback) using alarm cues from conspecifics (*P. amboinensis*, grey lines) or alarm cues from a closely-related heterospecific (*P. chrysurus*, black lines). The training took place in water containing cues from either healthy, live (solid lines) or dead-degraded (dashed lines) *Pocillopora damicornis* coral.

The Kaplan–Meier survival analysis revealed a significant survival difference among the four treatment groups (χ_3_^2^ = 22.3, *p* < 0.001, Fig. 3). Subsequent analysis revealed the survival of *P. amboinensis* trained with alarm cues from conspecifics had a lower survival if they were conditioned in dead coral than live coral environment (χ_1_^2^ = 16.7, *p* < 0.001), while no effect of coral was found on the survival on fish trained with *P. chrysurus* alarm cues (χ_1_^2^ = 0.14, *p* = 0.9). In live coral, however, juveniles trained with both types of alarm cues had similar survival (χ_1_^2^ = 1.0, *p* = 0.31).

## Discussion

The results of our study demonstrate that coral degradation causes severe learning impairment in Ambon damselfish. However, our study also demonstrated that learning redundancy appears to be built into the diverse coral reef communities minimizing these negative effects. While Ambon damselfish alarm cues were ineffective in degraded environments, manifesting in the failure to learn to recognize novel predators, the alarm cues of closely-related Whitetail damselfish maintain their potency in degraded habitats^12^ and facilitated learning in Ambon damselfish.

Damselfishes of the Indo-Pacific often form mixed species associations. As with any mixed species association, membership has advantages and disadvantages^36^. Living with other species often means additional competitors for both food and habitat^37^. Group membership may also change exposure to pathogens and parasites^38^. From the perspective of the Ambon damselfish, the learning of predators from whitetail damselfish may provide a distinct advantage when associating with them in degraded coral environments. However, it does not necessarily follow that the same advantage would occur in live coral environments because of this asymmetrical benefit. The antipredator advantage that the Ambon damselfish gains may come at a cost to the Whitetail damselfish, particularly if larger groups attract more predators^39^ and Whitetail damselfish are unable to use Ambon damselfish alarm cues to learn predators. Understanding social dynamics in degraded habitats, including the motivation of individuals and species to join or exclude individuals from joining multispecies groups, could be a fascinating area of future research and underscore the urgency in maintaining diversity^40^.

Coral reef fishes typically rely heavily on olfactory information not only for predator avoidance, but for other activities such as habitat recognition and settlement. For example, Lecchini et al.^41^ showed that 13 of 18 species of reef fishes use conspecific odours, rather than coral odour, to identify suitable settlement habitats. Some species such as *Chromis viridis*, are attracted to coral heads with conspecifics compared to uninhabited corals, but prefer uninhabited coral heads over corals with heterospecifics^42^. Others have shown that most reef fishes prefer water from reefs dominated by corals over reefs dominated by algae^43^. The widespread death of corals leading to algae dominated landscapes could, therefore, have implication for large scale settlement patterns. This may be exacerbated by exposure to pollutants. O’Connor et al.^44^ demonstrated that exposure to laterite sediments (red soil) altered fish olfactory responses, causing an attraction to dead corals over live corals.

The discipline of chemical ecology has been hampered by our inability to understand the chemistry behind the ecological interactions that we study^20,45,46^. This is amply evident in our current investigation. The two species have alarm cues that are structurally similar enough to allows for cross-species recognition^22,47^, which is a common trait among members of the family Pomacentridae^25^. Nevertheless, only one of our two species had alarm cues that were rendered ineffective by coral degradation. We do not know what is responsible for the breakdown of alarm cues, but McCormick et al.^13^ suggests that chemicals from cyanobacteria, *Okeania* sp., and diatoms, *Pseudo-nitzschia* sp. as well as the common red algae, *Galaxauria robusta*, prevented the alarm odour response. Untangling the chemistry behind the deactivation would be highly advantageous particularly if it provided us with a predictive framework to understand how other changes in water chemistry, such as those associated with ocean acidification or exposure to pollutants^26,48^, could influence responses to alarm cues.

The strength of our investigation on cross-species learning and the establishment of learning redundancy in mixed species groups is best exemplified by our behaviour and survival experiment in the wild. Ambon damselfish conditioned with heterospecific alarm cues in both live and dead coral, and those conditioned with conspecific alarm cues in live coral, were closer to their shelter, less bold and reduced feeding more than fish conditioned with conspecific alarm cues in dead coral. Perhaps most important, these behavioural effects lead to a significant survival benefit. The failure to quickly learn to identify predators through a single conditioning trial, as was done here, may be particularly important for juvenile reef fish which arrive at the reef with no knowledge of the predators that await them^49^. They must quickly catalogue which species are dangerous and which are not. This can be daunting task, given that predators can occur as singles and in mixed-species groups that can overcome prey defenses^50,51^.

Coral reef are tremendously diverse ecosystems, yet their keystone species, the coral themselves, may be highly vulnerable to anthropogenic change^52^. Given that we experienced the highest amount of ocean warming ever recorded in 2018, the situation on reefs is likely to worsen^53^. Studies such as ours, that seek to understand ecological redundancy of information, may provide insights into ways to protect these ecosystems^54^ such as prioritizing which species to target for special protection given their benefit to more vulnerable species in the community.

## Supplementary Information

Supplementary Information 1.

Supplementary Information 2.
