# Supplementary material for: Living in mixed species groups promotes predator learning in degraded habitats
Source: Sci Rep. 2021 Sep 29;11:19335. doi: 10.1038/s41598-021-98224-0 (PMC8481234; doi:10.1038/s41598-021-98224-0)
Supplement: Supplementary file 1 — Supplementary Information 1. [file 41598_2021_98224_MOESM1_ESM.docx]

**Living in mixed species groups promotes predator learning in degraded habitats**

Douglas P. Chivers, Mark I. McCormick, Eric P. Fakan, Randall P. Barry and Maud C.O. Ferrari

Supplementary Table 1. Baseline behaviour of the fish used in experiment 1. We failed to find any bias among treatment (3-way nested ANOVA, all p>0.5).

Df F P

Coral 1, 5.9 0.24 0.64

Conditioning 2, 92.1 0.76 0.47

Coral* Conditioning 2, 92.1 0.22 0.80

Conditioning tank 23, 70 0.43 0.99

Supplementary Table 2. Behavioural response of the fish used in experiment 1 (3-way nested ANOVA).

df F P

Coral 1, 13.07 13.3 0.003

Conditioning 2, 76.4 87.2 <0.001

Coral* Conditioning 2, 76.6 22.8 <0.001

Conditioning tank 23, 70 0.86 0.65

Supplementary Table 3. Behavioural response of the fish in experiment 2 (3-way nested ANOVA).

df F P

Coral 1, 6.0 37.4 <0.001

Conditioning 1, 6.1 11.25 0.015

Coral* Conditioning 1, 6.1 11.5 0.014

Conditioning tank 8, 87 1.5 0.17
